# Should we continue to use prediction tools to identify patients at risk of Candida spp. infection? If yes, why?

**DOI:** 10.1186/s13054-016-1521-0

**Published:** 2016-10-30

**Authors:** A. Cortegiani, V. Russotto, S. M. Raineri, G. Gregoretti, A. Giarratano

**Affiliations:** Department of Biopathology and Medical Biotechnologies (DIBIMED), Section of Anesthesia, Analgesia, Intensive Care and Emergency. Policlinico P. Giaccone, University of Palermo, Via del Vespro 129, 90127 Palermo, Italy

**Keywords:** Candida spp, Invasive fungal infections, Sepsis

We read with interest the article from Shanin et al. about the Fungal Infection Risk Evaluation (FIRE) study [1] aiming to ‘describe the incidence of IFD in UK critical care units and to develop and validate a clinical risk prediction tool to identify non-neutropenic, critically ill adult patients at risk of IFD’. The investigators should be congratulated for the way they collected a huge amount of data from 96 adult intensive care units (ICUs), managed the FIRE database, and developed and validated the risk model. However, they stated that the prediction model would help to identify patients who may benefit from antifungal prophylaxis and that a number of randomized controlled trials (RCTs) demonstrated a beneficial effect of antifungal prophylaxis and/or empiric treatment in terms of incidence of invasive fungal disease (IFD) and mortality. This statement is not supported by available evidence from RCTs. A recent Cochrane Systematic Review including 22 RCTs evaluating prophylaxis, pre-emptive, and empiric antifungal treatment with any antifungal drugs in 2761 non-neutropenic critically ill patients showed no significant effect on mortality (risk ratio (RR) 0.93, 95 % confidence interval (CI) 0.79 to 1.09) and a significant reduction in the risk of invasive fungal infection (IFI) (RR 0.57, 95 % CI 0.39 to 0.83) [2, 3]. In the subgroup analysis for type of intervention, antifungal prophylaxis was not associated with a significant mortality reduction but with a significant reduction of IFI [4]. This systematic review was the update of the one cited in the manuscript and published in 2006 including 12 RCT and 1606 patients.

Resistance to antifungals has become an increasingly burning clinical challenge. It concerns not only azoles but also the relatively new echinocandins [5]. Notably, the increasing use of antifungal drugs is linked to the development of resistance, whereas infections by resistant Candida spp. are linked to worse outcome [5]. How should predictive tools be used to identify patients at risk of IFI? What should we do after identifying a patient at risk of IFI while waiting for microbiology results? Maybe the answer should not be just giving antifungal prophylaxis as we did in the past. A more complex approach including the use of surrogate markers (e.g., beta-d-glucan), early use of other diagnostic test (e.g., multiplex polymerase chain reaction test, MALDI-TOF), source control, daily evaluation of risk factors, and, finally, in some cases the wise use of antifungal drugs according to local epidemiology may be answer. To simplify, antifungal stewardship is what we should implement to balance risk and benefit.

## Abbreviations

CI: Confidence interval
FIRE: Fungal Infection Risk Evaluation
ICU: Intensive care unit
IFD: Invasive fungal disease
IFI: Invasive fungal infection
MALDI-TOF: Matrix assisted laser desorption ionization-time of flight mass spectrometry
RCT: Randomized controlled trial
RR: Risk ratio

## References

[1] Shanin J, Allen EJ, Patel K, Muskett H, Harvey SE, Edgeworth J (2016). Predicting invasive fungal disease due to Candida species in non-neutropenic, critically ill, adult patients in United Kingdom critical care units. BMC Infect Dis.

[2] Cortegiani A, Russotto V, Maggiore A, Attanasio M, Naro AR, Raineri SM (2016). Antifungal agents for preventing fungal infections in non-neutropenic critically ill patients. Cochrane Database Syst Rev.

[3] Cortegiani A, Russotto V, Raineri SM, Giarratano A (2016). The paradox of the evidence about invasive fungal infection prevention. Crit Care.

[4] Cortegiani A, Russotto V, Raineri SM, Giarratano A (2016). Antifungal prophylaxis: update on an old strategy. Eur J Clin Microbiol Infect Dis.

[5] Arendrup MC, Perlin DS (2014). Echinocandin resistance: an emerging clinical problem?. Curr Opin Infect Dis.

